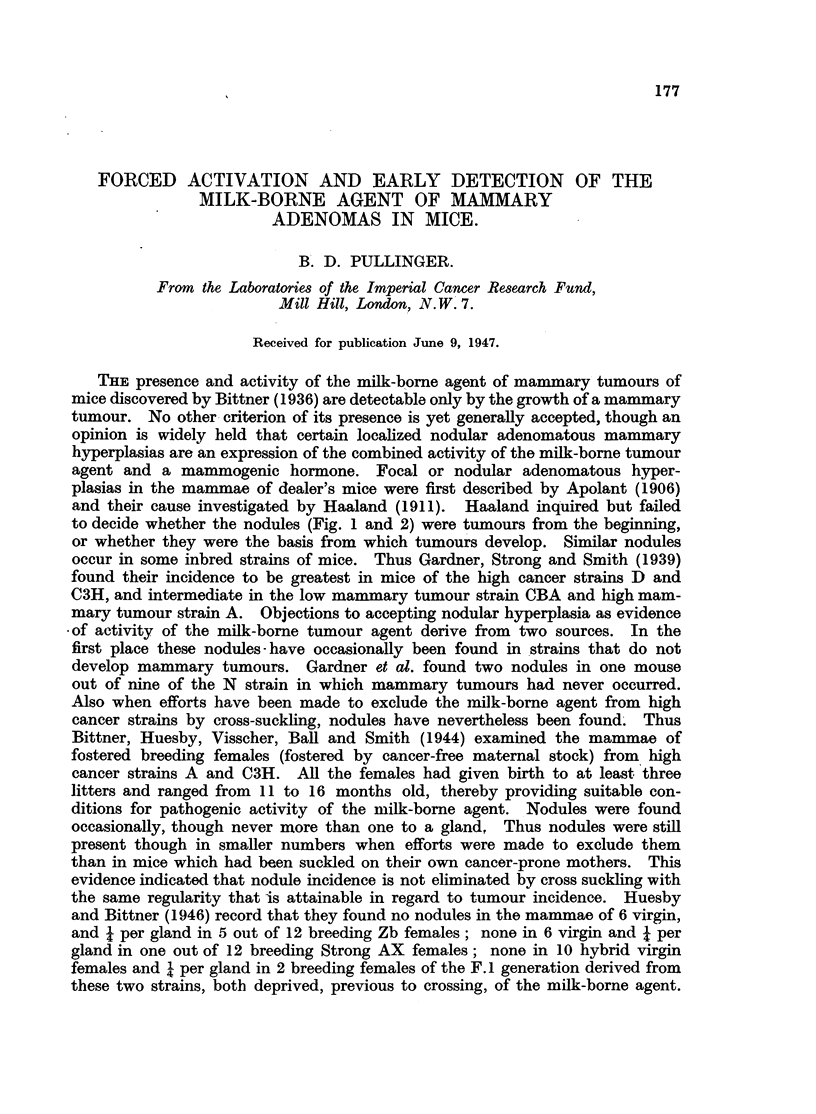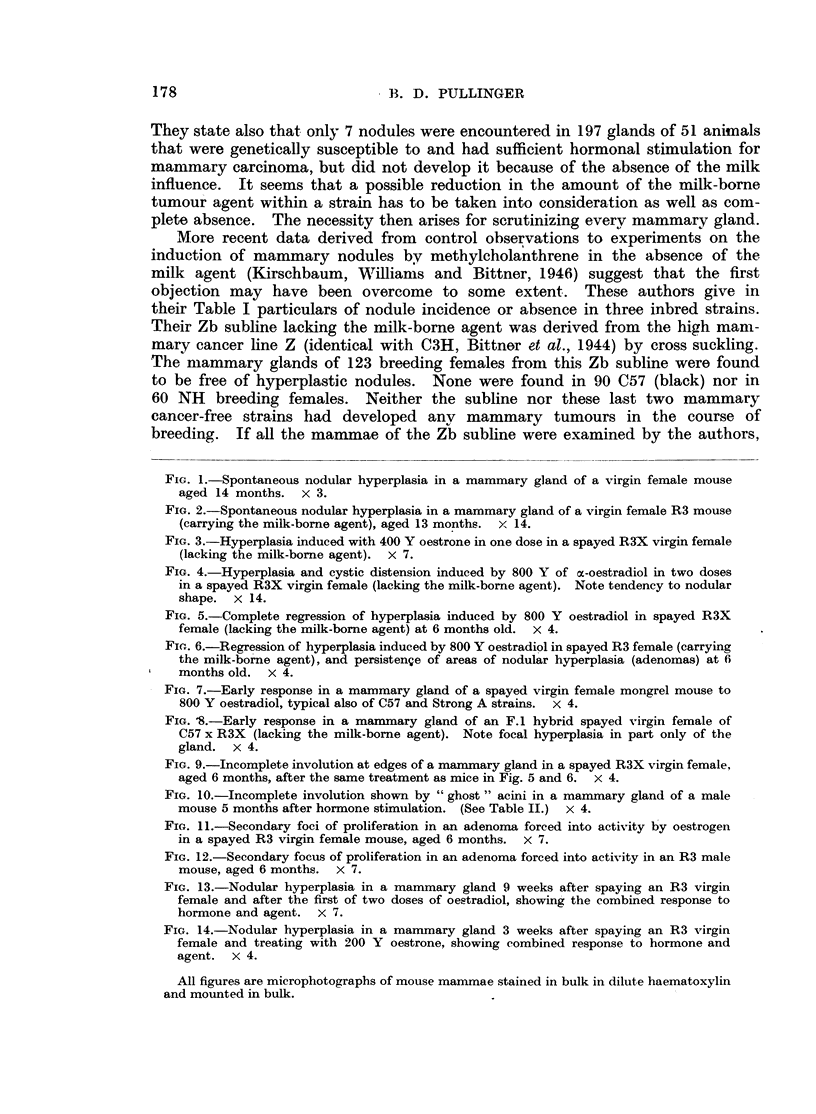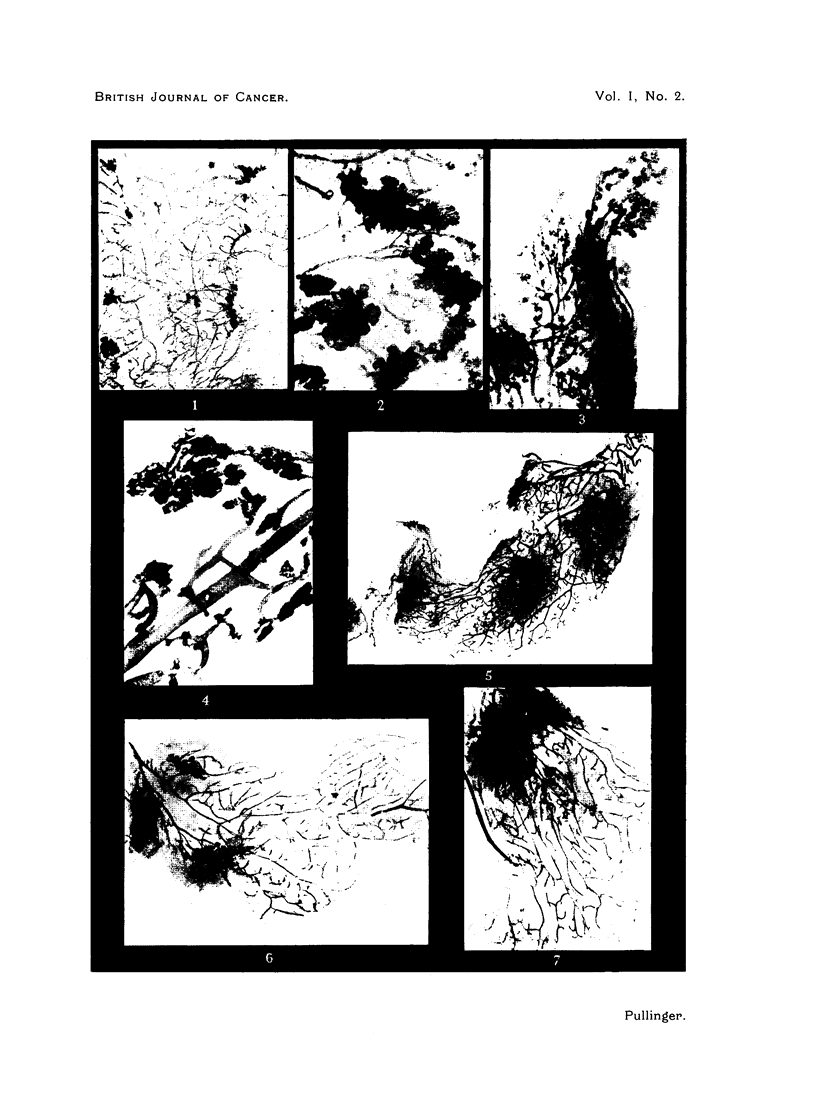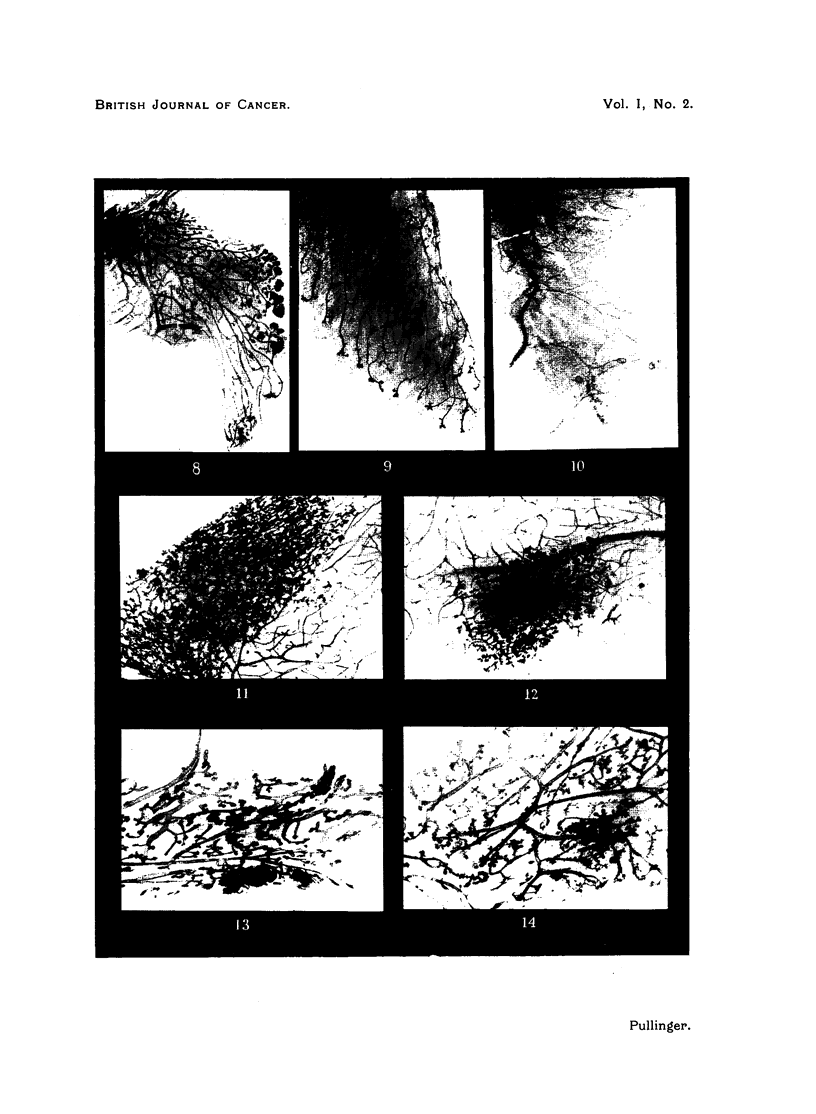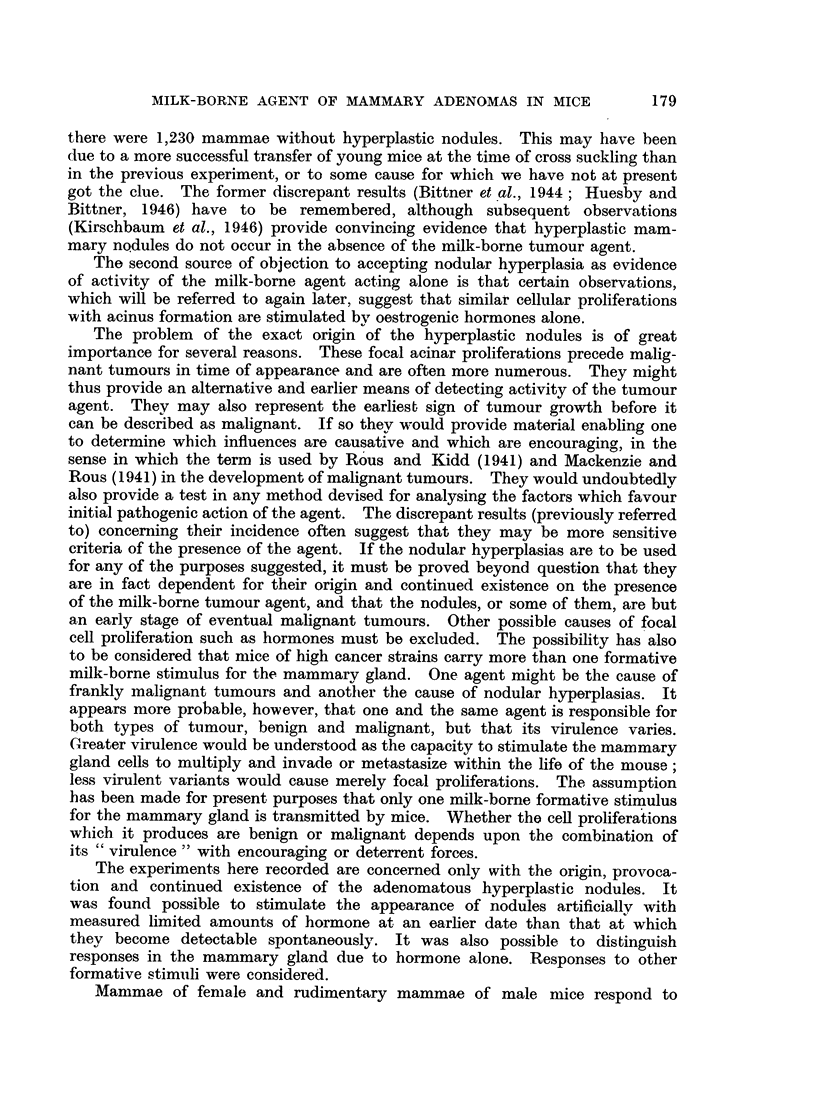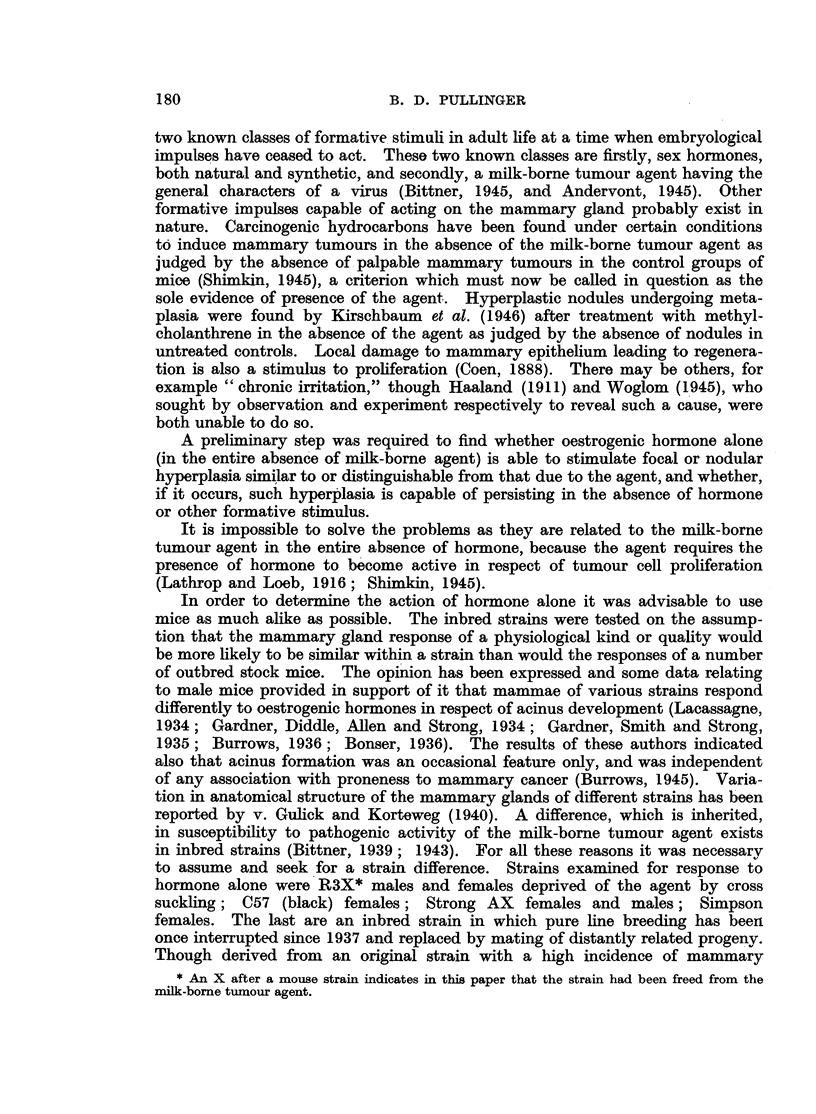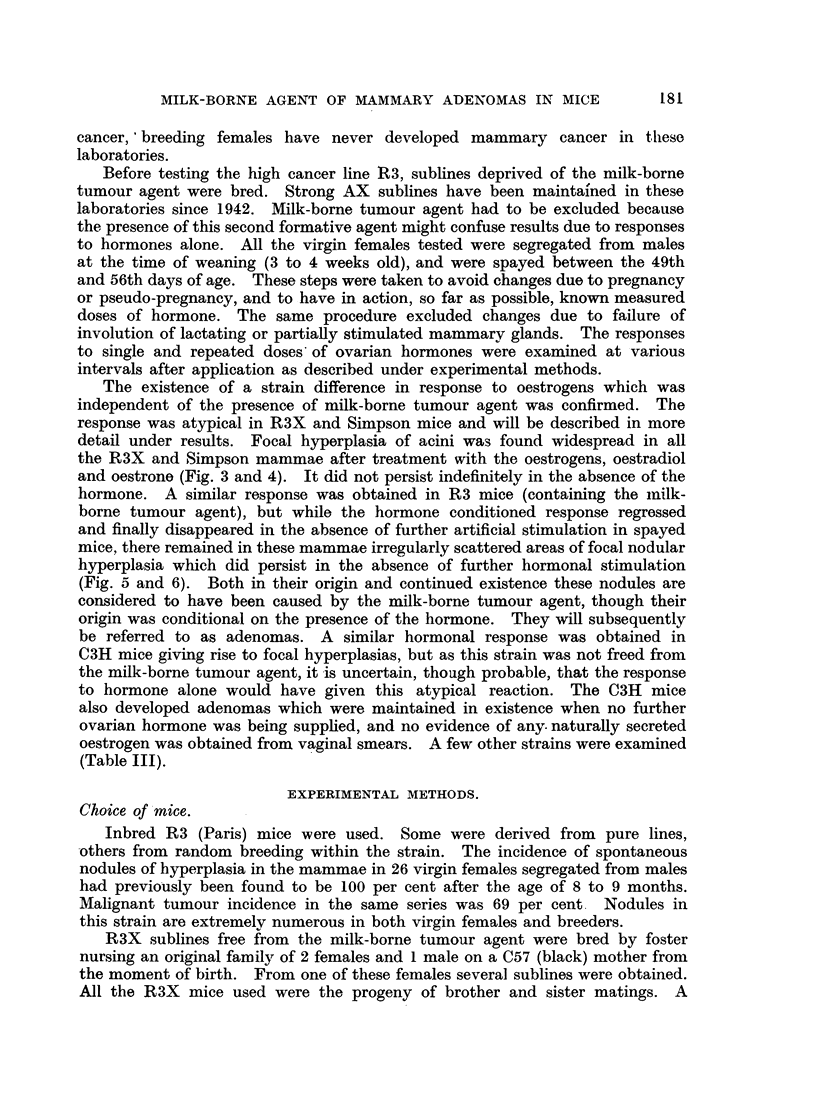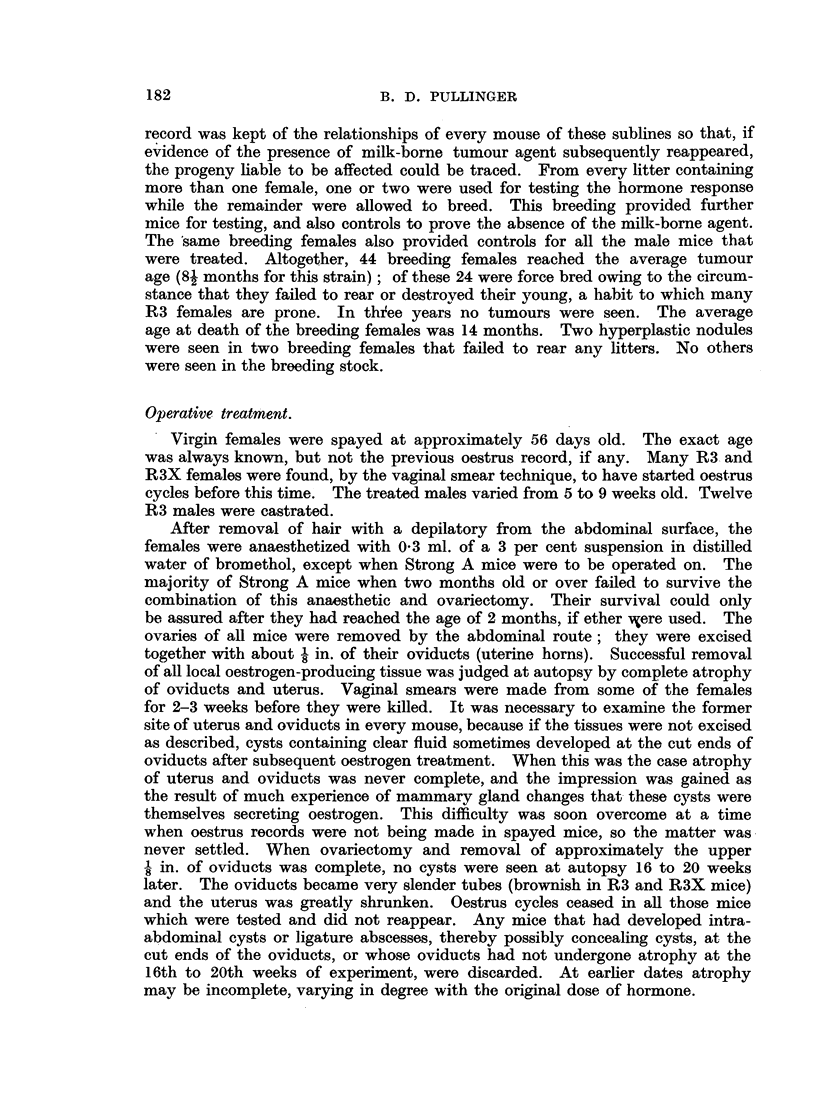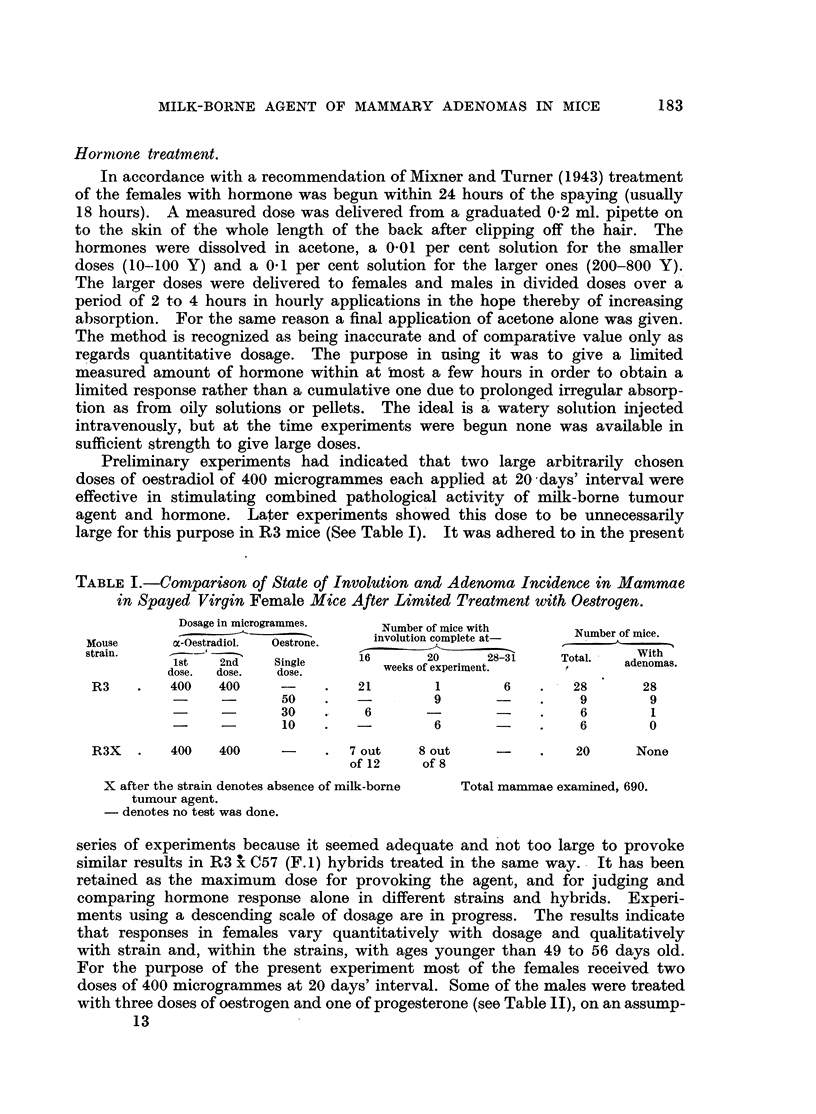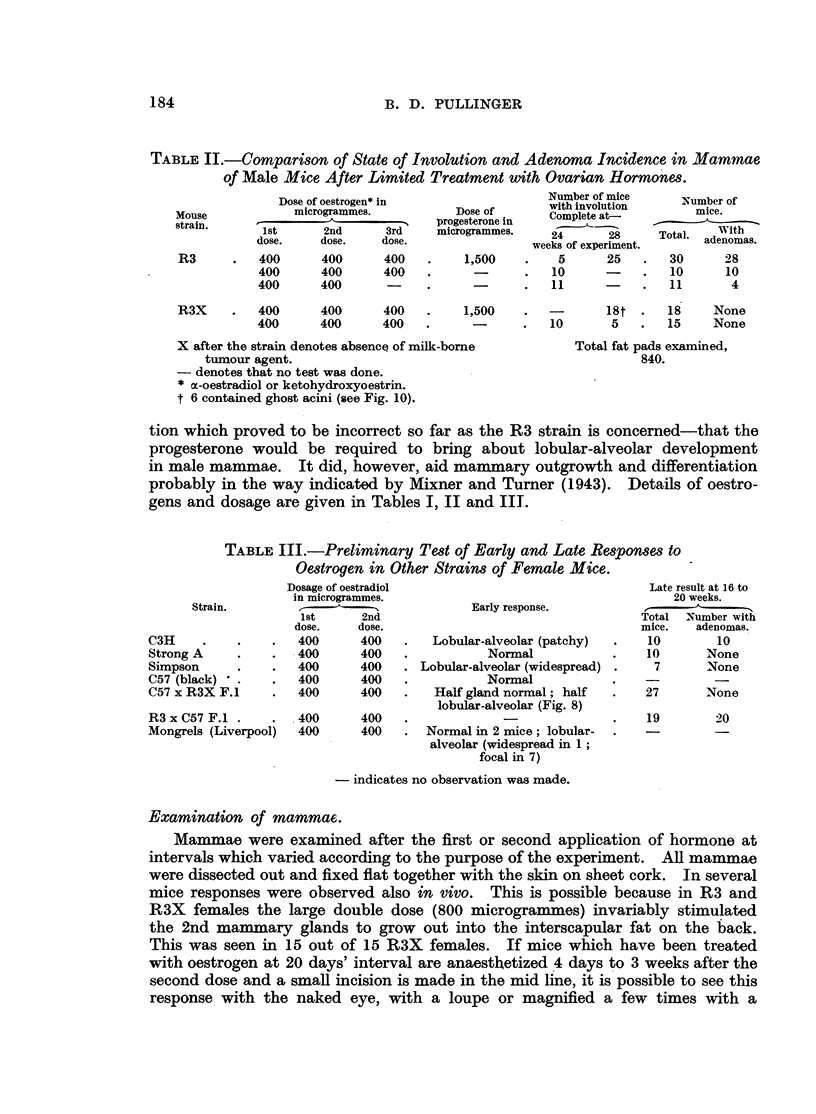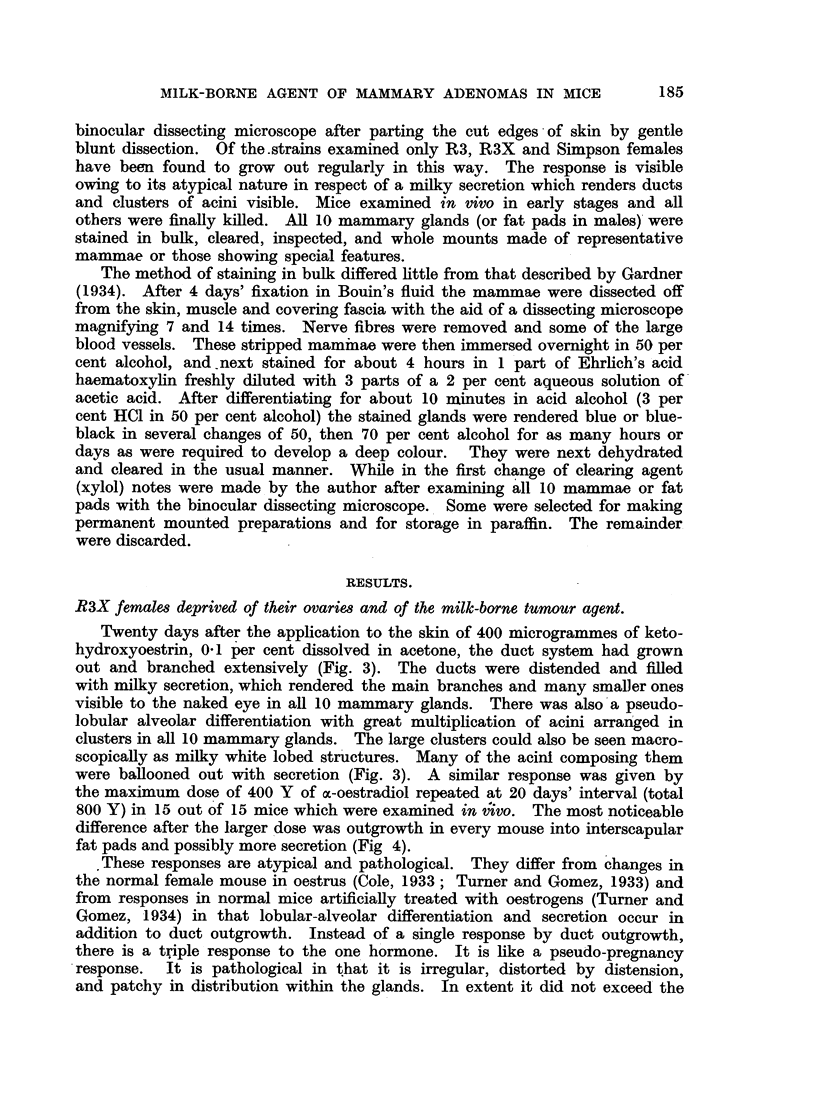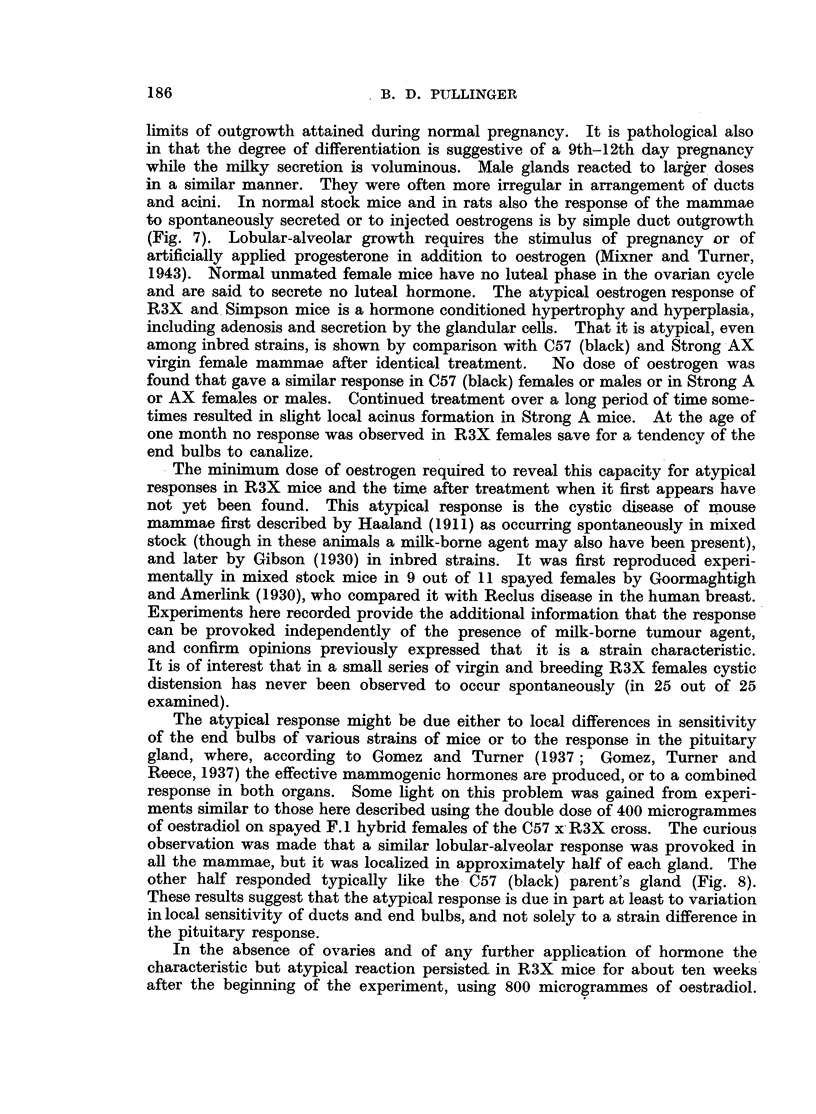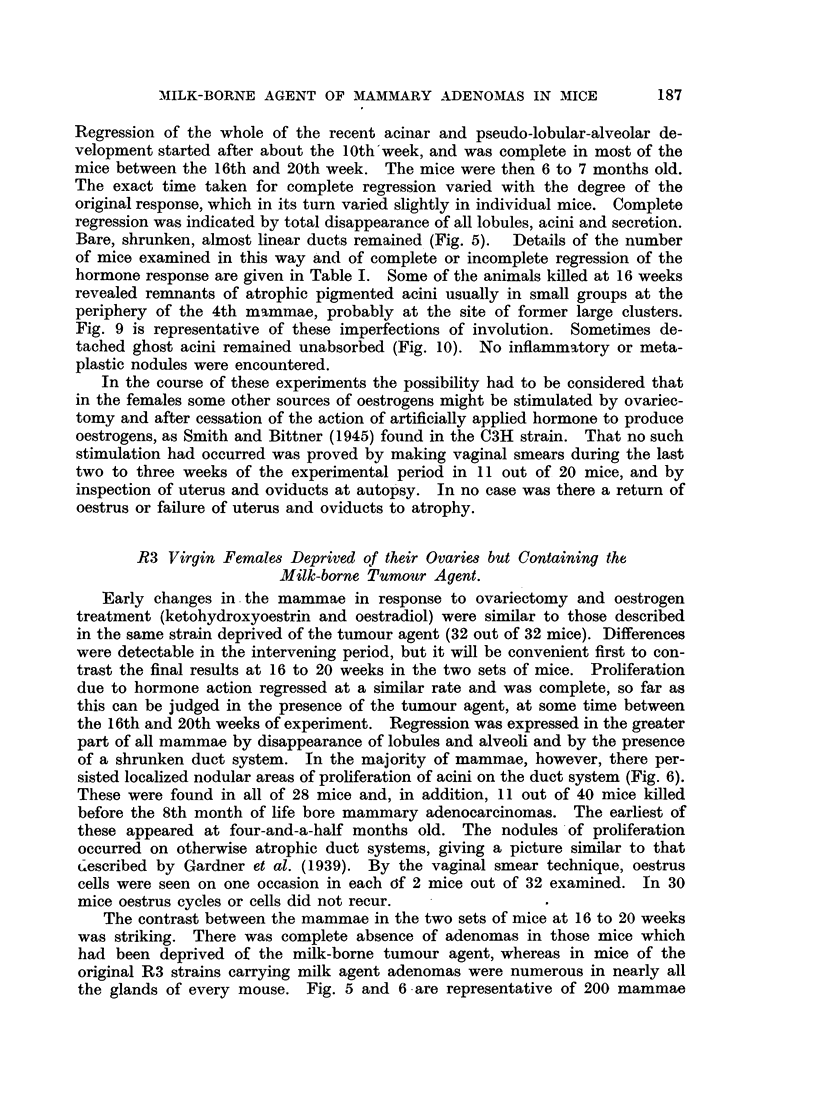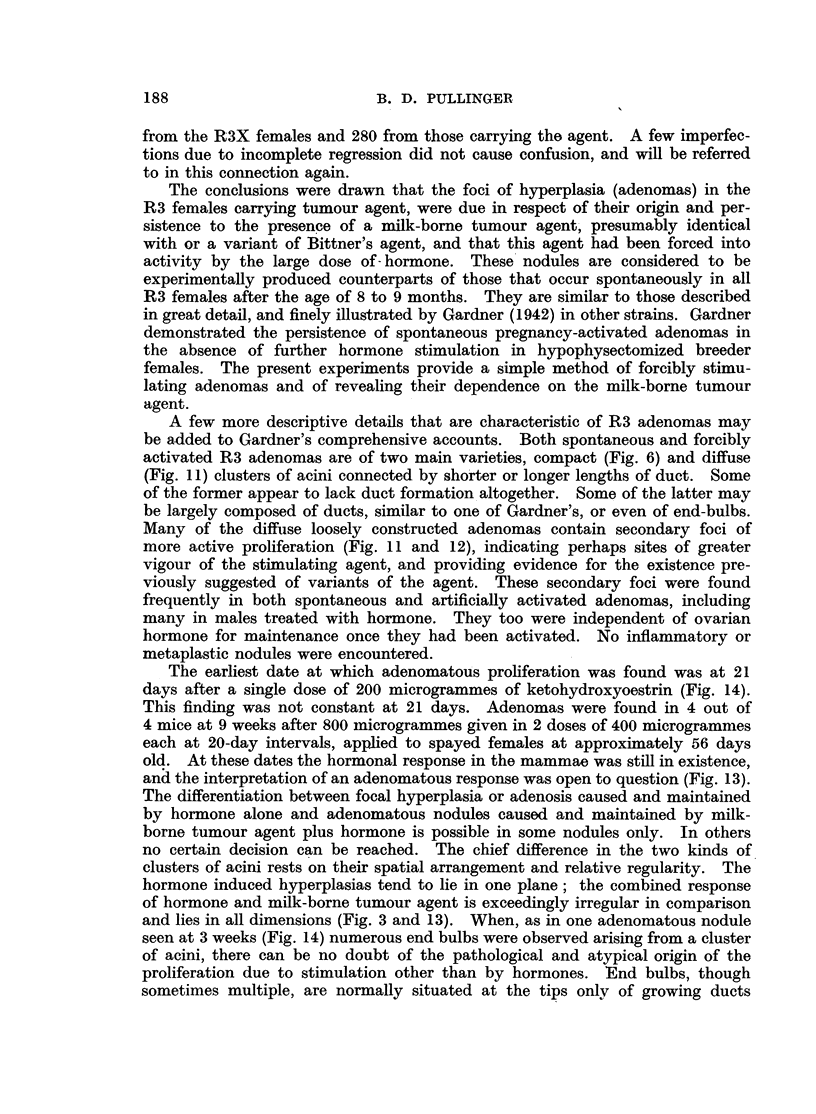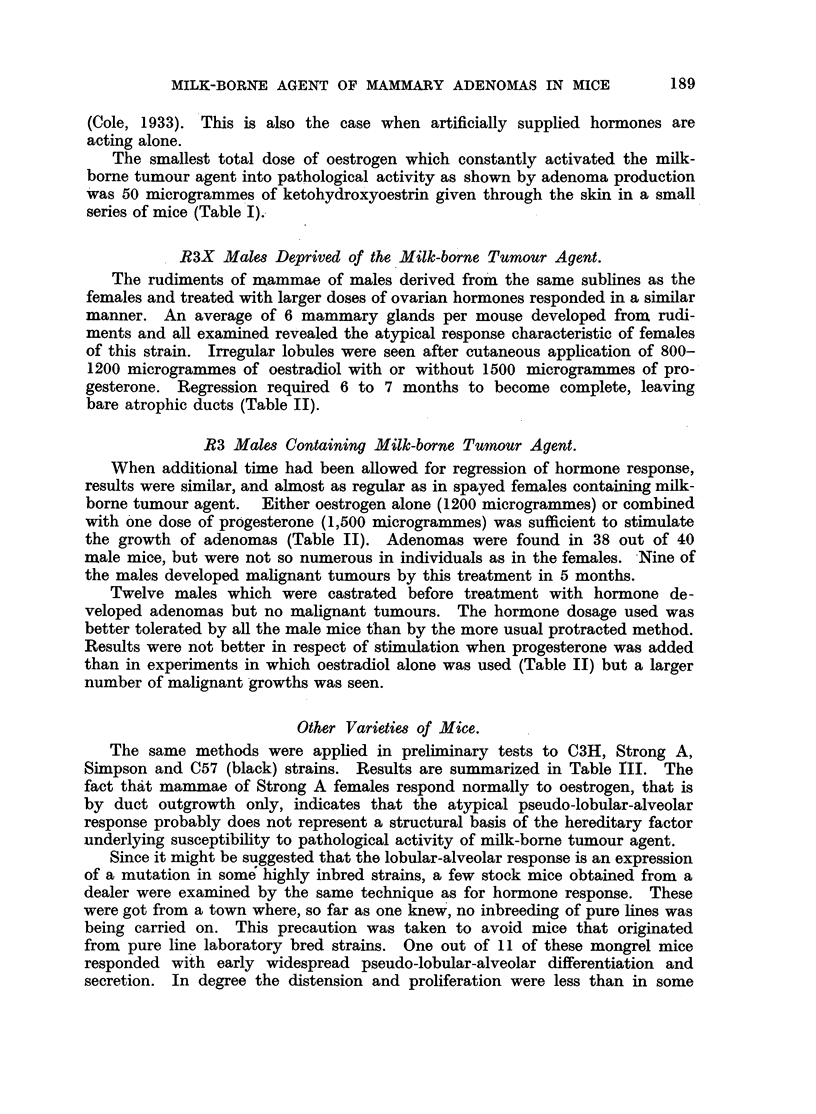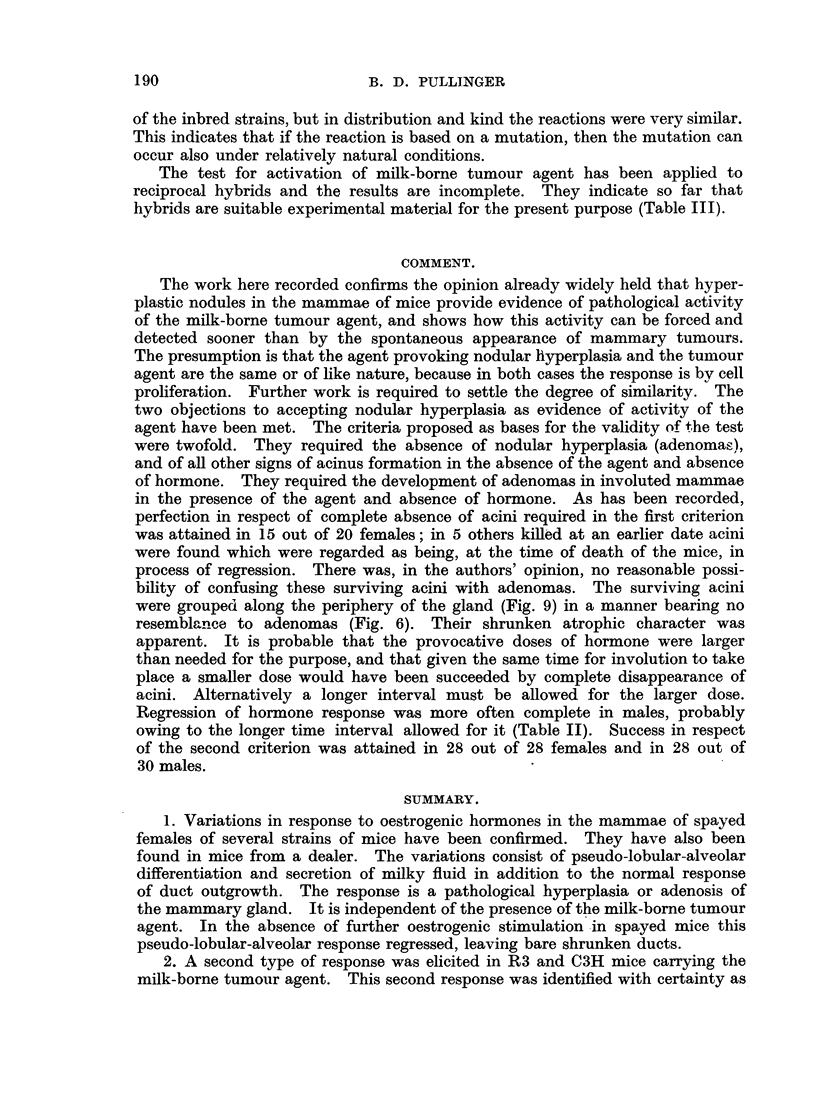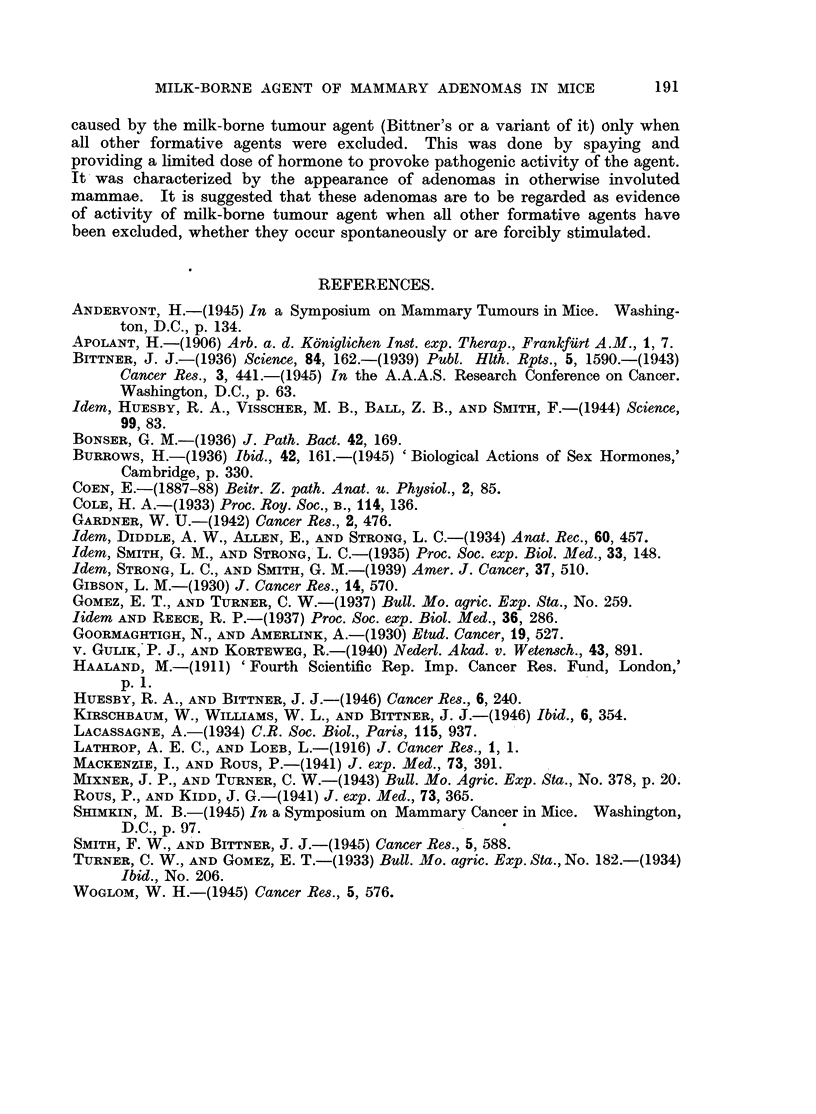# Forced Activation and Early Detection of the Milk-borne Agent of Mammary Adenomas in Mice

**DOI:** 10.1038/bjc.1947.21

**Published:** 1947-06

**Authors:** B. D. Pullinger

## Abstract

**Images:**


### FORCED ACTIVATION AND EARLY DETECTION OF THE MILK-BORNE AGENT OF MAMMARY ADENOMAS IN MICE.

B. D. PULLINGER.

*From the Laboratories of the Imperial Cancer Research Fund, Mill Hill, London, N.W. 7.*

Received for publication June 9, 1947.

THE presence and activity of the milk-borne agent of mammary tumours of mice discovered by Bittner (1936) are detectable only by the growth of a mammary tumour. No other criterion of its presence is yet generally accepted, though an opinion is widely held that certain localized nodular adenomatous mammary hyperplasias are an expression of the combined activity of the milk-borne tumour agent and a mammogenic hormone. Focal or nodular adenomatous hyperplasias in the mammae of dealer's mice were first described by Apolant (1906) and their cause investigated by Haaland (1911). Haaland inquired but failed to decide whether the nodules (Fig. 1 and 2) were tumours from the beginning, or whether they were the basis from which tumours develop. Similar nodules occur in some inbred strains of mice. Thus Gardner, Strong and Smith (1939) found their incidence to be greatest in mice of the high cancer strains D and C3H, and intermediate in the low mammary tumour strain CBA and high mammary tumour strain A. Objections to accepting nodular hyperplasia as evidence of activity of the milk-borne tumour agent derive from two sources. In the first place these nodules have occasionally been found in strains that do not develop mammary tumours. Gardner *et al.* found two nodules in one mouse out of nine of the N strain in which mammary tumours had never occurred. Also when efforts have been made to exclude the milk-borne agent from high cancer strains by cross-suckling, nodules have nevertheless been found. Thus Bittner, Huesby, Visscher, Ball and Smith (1944) examined the mammae of fostered breeding females (fostered by cancer-free maternal stock) from high cancer strains A and C3H. All the females had given birth to at least three litters and ranged from 11 to 16 months old, thereby providing suitable conditions for pathogenic activity of the milk-borne agent. Nodules were found occasionally, though never more than one to a gland, Thus nodules were still present though in smaller numbers when efforts were made to exclude them than in mice which had been suckled on their own cancer-prone mothers. This evidence indicated that nodule incidence is not eliminated by cross suckling with the same regularity that is attainable in regard to tumour incidence. Huesby and Bittner (1946) record that they found no nodules in the mammae of 6 virgin, and $\frac{1}{4}$ per gland in 5 out of 12 breeding Zb females ; none in 6 virgin and $\frac{1}{4}$ per gland in one out of 12 breeding Strong AX females ; none in 10 hybrid virgin females and $\frac{1}{4}$ per gland in 2 breeding females of the F.1 generation derived from these two strains, both deprived, previous to crossing, of the milk-borne agent.

They state also that only 7 nodules were encountered in 197 glands of 51 animals that were genetically susceptible to and had sufficient hormonal stimulation for mammary carcinoma, but did not develop it because of the absence of the milk influence. It seems that a possible reduction in the amount of the milk-borne tumour agent within a strain has to be taken into consideration as well as complete absence. The necessity then arises for scrutinizing every mammary gland.

More recent data derived from control observations to experiments on the induction of mammary nodules by methylcholanthrene in the absence of the milk agent (Kirschbaum, Williams and Bittner, 1946) suggest that the first objection may have been overcome to some extent. These authors give in their Table I particulars of nodule incidence or absence in three inbred strains. Their Zb subline lacking the milk-borne agent was derived from the high mammary cancer line Z (identical with C3H, Bittner *et al.*, 1944) by cross suckling. The mammary glands of 123 breeding females from this Zb subline were found to be free of hyperplastic nodules. None were found in 90 C57 (black) nor in 60 NH breeding females. Neither the subline nor these last two mammary cancer-free strains had developed any mammary tumours in the course of breeding. If all the mammae of the Zb subline were examined by the authors,

---

FIG. 1.—Spontaneous nodular hyperplasia in a mammary gland of a virgin female mouse aged 14 months. × 3.

FIG. 2.—Spontaneous nodular hyperplasia in a mammary gland of a virgin female R3 mouse (carrying the milk-borne agent), aged 13 months. × 14.

FIG. 3.—Hyperplasia induced with 400 Y oestrone in one dose in a spayed R3X virgin female (lacking the milk-borne agent). × 7.

FIG. 4.—Hyperplasia and cystic distension induced by 800 Y of α-oestradiol in two doses in a spayed R3X virgin female (lacking the milk-borne agent). Note tendency to nodular shape. × 14.

FIG. 5.—Complete regression of hyperplasia induced by 800 Y oestradiol in spayed R3X female (lacking the milk-borne agent) at 6 months old. × 4.

FIG. 6.—Regression of hyperplasia induced by 800 Y oestradiol in spayed R3 female (carrying the milk-borne agent), and persistençe of areas of nodular hyperplasia (adenomas) at 6 months old. × 4.

FIG. 7.—Early response in a mammary gland of a spayed virgin female mongrel mouse to 800 Y oestradiol, typical also of C57 and Strong A strains. × 4.

FIG. 8.—Early response in a mammary gland of an F.1 hybrid spayed virgin female of C57 x R3X (lacking the milk-borne agent). Note focal hyperplasia in part only of the gland. × 4.

FIG. 9.—Incomplete involution at edges of a mammary gland in a spayed R3X virgin female, aged 6 months, after the same treatment as mice in Fig. 5 and 6. × 4.

FIG. 10.—Incomplete involution shown by " ghost " acini in a mammary gland of a male mouse 5 months after hormone stimulation. (See Table II.) × 4.

FIG. 11.—Secondary foci of proliferation in an adenoma forced into activity by oestrogen in a spayed R3 virgin female mouse, aged 6 months. × 7.

FIG. 12.—Secondary focus of proliferation in an adenoma forced into activity in an R3 male mouse, aged 6 months. × 7.

FIG. 13.—Nodular hyperplasia in a mammary gland 9 weeks after spaying an R3 virgin female and after the first of two doses of oestradiol, showing the combined response to hormone and agent. × 7.

FIG. 14.—Nodular hyperplasia in a mammary gland 3 weeks after spaying an R3 virgin female and treating with 200 Y oestrone, showing combined response to hormone and agent. × 4.

All figures are microphotographs of mouse mammae stained in bulk in dilute haematoxylin and mounted in bulk.

1

2

3

4

5

6

7

Pullinger.

8

9

10

11

12

13

14

there were 1,230 mammae without hyperplastic nodules. This may have been due to a more successful transfer of young mice at the time of cross suckling than in the previous experiment, or to some cause for which we have not at present got the clue. The former discrepant results (Bittner *et al.*, 1944 ; Huesby and Bittner, 1946) have to be remembered, although subsequent observations (Kirschbaum *et al.*, 1946) provide convincing evidence that hyperplastic mammary nodules do not occur in the absence of the milk-borne tumour agent.

The second source of objection to accepting nodular hyperplasia as evidence of activity of the milk-borne agent acting alone is that certain observations, which will be referred to again later, suggest that similar cellular proliferations with acinus formation are stimulated by oestrogenic hormones alone.

The problem of the exact origin of the hyperplastic nodules is of great importance for several reasons. These focal acinar proliferations precede malignant tumours in time of appearance and are often more numerous. They might thus provide an alternative and earlier means of detecting activity of the tumour agent. They may also represent the earliest sign of tumour growth before it can be described as malignant. If so they would provide material enabling one to determine which influences are causative and which are encouraging, in the sense in which the term is used by Rous and Kidd (1941) and Mackenzie and Rous (1941) in the development of malignant tumours. They would undoubtedly also provide a test in any method devised for analysing the factors which favour initial pathogenic action of the agent. The discrepant results (previously referred to) concerning their incidence often suggest that they may be more sensitive criteria of the presence of the agent. If the nodular hyperplasias are to be used for any of the purposes suggested, it must be proved beyond question that they are in fact dependent for their origin and continued existence on the presence of the milk-borne tumour agent, and that the nodules, or some of them, are but an early stage of eventual malignant tumours. Other possible causes of focal cell proliferation such as hormones must be excluded. The possibility has also to be considered that mice of high cancer strains carry more than one formative milk-borne stimulus for the mammary gland. One agent might be the cause of frankly malignant tumours and another the cause of nodular hyperplasias. It appears more probable, however, that one and the same agent is responsible for both types of tumour, benign and malignant, but that its virulence varies. Greater virulence would be understood as the capacity to stimulate the mammary gland cells to multiply and invade or metastasize within the life of the mouse ; less virulent variants would cause merely focal proliferations. The assumption has been made for present purposes that only one milk-borne formative stimulus for the mammary gland is transmitted by mice. Whether the cell proliferations which it produces are benign or malignant depends upon the combination of its " virulence " with encouraging or deterrent forces.

The experiments here recorded are concerned only with the origin, provocation and continued existence of the adenomatous hyperplastic nodules. It was found possible to stimulate the appearance of nodules artificially with measured limited amounts of hormone at an earlier date than that at which they become detectable spontaneously. It was also possible to distinguish responses in the mammary gland due to hormone alone. Responses to other formative stimuli were considered.

Mammae of female and rudimentary mammae of male mice respond to two known classes of formative stimuli in adult life at a time when embryological impulses have ceased to act. These two known classes are firstly, sex hormones, both natural and synthetic, and secondly, a milk-borne tumour agent having the general characters of a virus (Bittner, 1945, and Andervont, 1945). Other formative impulses capable of acting on the mammary gland probably exist in nature. Carcinogenic hydrocarbons have been found under certain conditions to induce mammary tumours in the absence of the milk-borne tumour agent as judged by the absence of palpable mammary tumours in the control groups of mice (Shimkin, 1945), a criterion which must now be called in question as the sole evidence of presence of the agent. Hyperplastic nodules undergoing metaplasia were found by Kirschbaum *et al.* (1946) after treatment with methylcholanthrene in the absence of the agent as judged by the absence of nodules in untreated controls. Local damage to mammary epithelium leading to regeneration is also a stimulus to proliferation (Coen, 1888). There may be others, for example "chronic irritation," though Haaland (1911) and Woglom (1945), who sought by observation and experiment respectively to reveal such a cause, were both unable to do so.

A preliminary step was required to find whether oestrogenic hormone alone (in the entire absence of milk-borne agent) is able to stimulate focal or nodular hyperplasia similar to or distinguishable from that due to the agent, and whether, if it occurs, such hyperplasia is capable of persisting in the absence of hormone or other formative stimulus.

It is impossible to solve the problems as they are related to the milk-borne tumour agent in the entire absence of hormone, because the agent requires the presence of hormone to become active in respect of tumour cell proliferation (Lathrop and Loeb, 1916; Shimkin, 1945).

In order to determine the action of hormone alone it was advisable to use mice as much alike as possible. The inbred strains were tested on the assumption that the mammary gland response of a physiological kind or quality would be more likely to be similar within a strain than would the responses of a number of outbred stock mice. The opinion has been expressed and some data relating to male mice provided in support of it that mammae of various strains respond differently to oestrogenic hormones in respect of acinus development (Lacassagne, 1934; Gardner, Diddle, Allen and Strong, 1934; Gardner, Smith and Strong, 1935; Burrows, 1936; Bonser, 1936). The results of these authors indicated also that acinus formation was an occasional feature only, and was independent of any association with proneness to mammary cancer (Burrows, 1945). Variation in anatomical structure of the mammary glands of different strains has been reported by v. Gulick and Korteweg (1940). A difference, which is inherited, in susceptibility to pathogenic activity of the milk-borne tumour agent exists in inbred strains (Bittner, 1939; 1943). For all these reasons it was necessary to assume and seek for a strain difference. Strains examined for response to hormone alone were R3X* males and females deprived of the agent by cross suckling; C57 (black) females; Strong AX females and males; Simpson females. The last are an inbred strain in which pure line breeding has been once interrupted since 1937 and replaced by mating of distantly related progeny. Though derived from an original strain with a high incidence of mammary

* An X after a mouse strain indicates in this paper that the strain had been freed from the milk-borne tumour agent.

cancer, breeding females have never developed mammary cancer in these laboratories.

Before testing the high cancer line R3, sublines deprived of the milk-borne tumour agent were bred. Strong AX sublines have been maintained in these laboratories since 1942. Milk-borne tumour agent had to be excluded because the presence of this second formative agent might confuse results due to responses to hormones alone. All the virgin females tested were segregated from males at the time of weaning (3 to 4 weeks old), and were spayed between the 49th and 56th days of age. These steps were taken to avoid changes due to pregnancy or pseudo-pregnancy, and to have in action, so far as possible, known measured doses of hormone. The same procedure excluded changes due to failure of involution of lactating or partially stimulated mammary glands. The responses to single and repeated doses of ovarian hormones were examined at various intervals after application as described under experimental methods.

The existence of a strain difference in response to oestrogens which was independent of the presence of milk-borne tumour agent was confirmed. The response was atypical in R3X and Simpson mice and will be described in more detail under results. Focal hyperplasia of acini was found widespread in all the R3X and Simpson mammae after treatment with the oestrogens, oestradiol and oestrone (Fig. 3 and 4). It did not persist indefinitely in the absence of the hormone. A similar response was obtained in R3 mice (containing the milk-borne tumour agent), but while the hormone conditioned response regressed and finally disappeared in the absence of further artificial stimulation in spayed mice, there remained in these mammae irregularly scattered areas of focal nodular hyperplasia which did persist in the absence of further hormonal stimulation (Fig. 5 and 6). Both in their origin and continued existence these nodules are considered to have been caused by the milk-borne tumour agent, though their origin was conditional on the presence of the hormone. They will subsequently be referred to as adenomas. A similar hormonal response was obtained in C3H mice giving rise to focal hyperplasias, but as this strain was not freed from the milk-borne tumour agent, it is uncertain, though probable, that the response to hormone alone would have given this atypical reaction. The C3H mice also developed adenomas which were maintained in existence when no further ovarian hormone was being supplied, and no evidence of any naturally secreted oestrogen was obtained from vaginal smears. A few other strains were examined (Table III).

### EXPERIMENTAL METHODS.

*Choice of mice.*

Inbred R3 (Paris) mice were used. Some were derived from pure lines, others from random breeding within the strain. The incidence of spontaneous nodules of hyperplasia in the mammae in 26 virgin females segregated from males had previously been found to be 100 per cent after the age of 8 to 9 months. Malignant tumour incidence in the same series was 69 per cent. Nodules in this strain are extremely numerous in both virgin females and breeders.

R3X sublines free from the milk-borne tumour agent were bred by foster nursing an original family of 2 females and 1 male on a C57 (black) mother from the moment of birth. From one of these females several sublines were obtained. All the R3X mice used were the progeny of brother and sister matings. A

record was kept of the relationships of every mouse of these sublines so that, if evidence of the presence of milk-borne tumour agent subsequently reappeared, the progeny liable to be affected could be traced. From every litter containing more than one female, one or two were used for testing the hormone response while the remainder were allowed to breed. This breeding provided further mice for testing, and also controls to prove the absence of the milk-borne agent. The same breeding females also provided controls for all the male mice that were treated. Altogether, 44 breeding females reached the average tumour age ($8\frac{1}{2}$ months for this strain); of these 24 were force bred owing to the circumstance that they failed to rear or destroyed their young, a habit to which many R3 females are prone. In three years no tumours were seen. The average age at death of the breeding females was 14 months. Two hyperplastic nodules were seen in two breeding females that failed to rear any litters. No others were seen in the breeding stock.

*Operative treatment.*

Virgin females were spayed at approximately 56 days old. The exact age was always known, but not the previous oestrus record, if any. Many R3 and R3X females were found, by the vaginal smear technique, to have started oestrus cycles before this time. The treated males varied from 5 to 9 weeks old. Twelve R3 males were castrated.

After removal of hair with a depilatory from the abdominal surface, the females were anaesthetized with 0·3 ml. of a 3 per cent suspension in distilled water of bromethol, except when Strong A mice were to be operated on. The majority of Strong A mice when two months old or over failed to survive the combination of this anaesthetic and ovariectomy. Their survival could only be assured after they had reached the age of 2 months, if ether were used. The ovaries of all mice were removed by the abdominal route; they were excised together with about $\frac{1}{8}$ in. of their oviducts (uterine horns). Successful removal of all local oestrogen-producing tissue was judged at autopsy by complete atrophy of oviducts and uterus. Vaginal smears were made from some of the females for 2–3 weeks before they were killed. It was necessary to examine the former site of uterus and oviducts in every mouse, because if the tissues were not excised as described, cysts containing clear fluid sometimes developed at the cut ends of oviducts after subsequent oestrogen treatment. When this was the case atrophy of uterus and oviducts was never complete, and the impression was gained as the result of much experience of mammary gland changes that these cysts were themselves secreting oestrogen. This difficulty was soon overcome at a time when oestrus records were not being made in spayed mice, so the matter was never settled. When ovariectomy and removal of approximately the upper $\frac{1}{8}$ in. of oviducts was complete, no cysts were seen at autopsy 16 to 20 weeks later. The oviducts became very slender tubes (brownish in R3 and R3X mice) and the uterus was greatly shrunken. Oestrus cycles ceased in all those mice which were tested and did not reappear. Any mice that had developed intra-abdominal cysts or ligature abscesses, thereby possibly concealing cysts, at the cut ends of the oviducts, or whose oviducts had not undergone atrophy at the 16th to 20th weeks of experiment, were discarded. At earlier dates atrophy may be incomplete, varying in degree with the original dose of hormone.

*Hormone treatment.*

In accordance with a recommendation of Mixner and Turner (1943) treatment of the females with hormone was begun within 24 hours of the spaying (usually 18 hours). A measured dose was delivered from a graduated 0·2 ml. pipette on to the skin of the whole length of the back after clipping off the hair. The hormones were dissolved in acetone, a 0·01 per cent solution for the smaller doses (10–100 Y) and a 0·1 per cent solution for the larger ones (200–800 Y). The larger doses were delivered to females and males in divided doses over a period of 2 to 4 hours in hourly applications in the hope thereby of increasing absorption. For the same reason a final application of acetone alone was given. The method is recognized as being inaccurate and of comparative value only as regards quantitative dosage. The purpose in using it was to give a limited measured amount of hormone within at most a few hours in order to obtain a limited response rather than a cumulative one due to prolonged irregular absorption as from oily solutions or pellets. The ideal is a watery solution injected intravenously, but at the time experiments were begun none was available in sufficient strength to give large doses.

Preliminary experiments had indicated that two large arbitrarily chosen doses of oestradiol of 400 microgrammes each applied at 20 days' interval were effective in stimulating combined pathological activity of milk-borne tumour agent and hormone. Later experiments showed this dose to be unnecessarily large for this purpose in R3 mice (See Table I). It was adhered to in the present

TABLE I.—*Comparison of State of Involution and Adenoma Incidence in Mammae in Spayed Virgin* Female *Mice After Limited Treatment with Oestrogen.*

| Mouse strain. | Dosage in microgrammes. α-Oestradiol. 1st dose. | 2nd dose. | Oestrone. Single dose. | Number of mice with involution complete at— 16 weeks of experiment. | 20 | 28–31 | Number of mice. Total. | With adenomas. |
|---|---|---|---|---|---|---|---|---|
| R3 | 400 | 400 | — | 21 | 1 | 6 | 28 | 28 |
| | — | — | 50 | — | 9 | — | 9 | 9 |
| | — | — | 30 | 6 | — | — | 6 | 1 |
| | — | — | 10 | — | 6 | — | 6 | 0 |
| R3X | 400 | 400 | — | 7 out of 12 | 8 out of 8 | — | 20 | None |

X after the strain denotes absence of milk-borne tumour agent.
— denotes no test was done.

Total mammae examined, 690.

series of experiments because it seemed adequate and not too large to provoke similar results in R3 x C57 (F.1) hybrids treated in the same way. It has been retained as the maximum dose for provoking the agent, and for judging and comparing hormone response alone in different strains and hybrids. Experiments using a descending scale of dosage are in progress. The results indicate that responses in females vary quantitatively with dosage and qualitatively with strain and, within the strains, with ages younger than 49 to 56 days old. For the purpose of the present experiment most of the females received two doses of 400 microgrammes at 20 days' interval. Some of the males were treated with three doses of oestrogen and one of progesterone (see Table II), on an assump-

TABLE II.—*Comparison of State of Involution and Adenoma Incidence in Mammae of* Male *Mice After Limited Treatment with Ovarian Hormones.*

| Mouse strain. | Dose of oestrogen* in microgrammes. | | | Dose of progesterone in microgrammes. | Number of mice with involution Complete at— | | Number of mice. | |
|---|---|---|---|---|---|---|---|---|
| | 1st dose. | 2nd dose. | 3rd dose. | | 24 weeks of experiment. | 28 weeks of experiment. | Total. | With adenomas. |
| R3 | 400 | 400 | 400 | 1,500 | 5 | 25 | 30 | 28 |
| | 400 | 400 | 400 | — | 10 | — | 10 | 10 |
| | 400 | 400 | — | — | 11 | — | 11 | 4 |
| R3X | 400 | 400 | 400 | 1,500 | — | 18† | 18 | None |
| | 400 | 400 | 400 | — | 10 | 5 | 15 | None |

X after the strain denotes absence of milk-borne tumour agent.
— denotes that no test was done.
* α-oestradiol or ketohydroxyoestrin.
† 6 contained ghost acini (see Fig. 10).

Total fat pads examined, 840.

tion which proved to be incorrect so far as the R3 strain is concerned—that the progesterone would be required to bring about lobular-alveolar development in male mammae. It did, however, aid mammary outgrowth and differentiation probably in the way indicated by Mixner and Turner (1943). Details of oestrogens and dosage are given in Tables I, II and III.

TABLE III.—*Preliminary Test of Early and Late Responses to Oestrogen in Other Strains of Female Mice.*

| Strain. | Dosage of oestradiol in microgrammes. | | Early response. | Late result at 16 to 20 weeks. | |
|---|---|---|---|---|---|
| | 1st dose. | 2nd dose. | | Total mice. | Number with adenomas. |
| C3H | 400 | 400 | Lobular-alveolar (patchy) | 10 | 10 |
| Strong A | 400 | 400 | Normal | 10 | None |
| Simpson | 400 | 400 | Lobular-alveolar (widespread) | 7 | None |
| C57 (black) | 400 | 400 | Normal | — | — |
| C57 x R3X F.1 | 400 | 400 | Half gland normal; half lobular-alveolar (Fig. 8) | 27 | None |
| R3 x C57 F.1 | 400 | 400 | — | 19 | 20 |
| Mongrels (Liverpool) | 400 | 400 | Normal in 2 mice; lobular-alveolar (widespread in 1; focal in 7) | — | — |

— indicates no observation was made.

*Examination of mammae.*

Mammae were examined after the first or second application of hormone at intervals which varied according to the purpose of the experiment. All mammae were dissected out and fixed flat together with the skin on sheet cork. In several mice responses were observed also *in vivo.* This is possible because in R3 and R3X females the large double dose (800 microgrammes) invariably stimulated the 2nd mammary glands to grow out into the interscapular fat on the back. This was seen in 15 out of 15 R3X females. If mice which have been treated with oestrogen at 20 days' interval are anaesthetized 4 days to 3 weeks after the second dose and a small incision is made in the mid line, it is possible to see this response with the naked eye, with a loupe or magnified a few times with a binocular dissecting microscope after parting the cut edges of skin by gentle blunt dissection. Of the strains examined only R3, R3X and Simpson females have been found to grow out regularly in this way. The response is visible owing to its atypical nature in respect of a milky secretion which renders ducts and clusters of acini visible. Mice examined *in vivo* in early stages and all others were finally killed. All 10 mammary glands (or fat pads in males) were stained in bulk, cleared, inspected, and whole mounts made of representative mammae or those showing special features.

The method of staining in bulk differed little from that described by Gardner (1934). After 4 days' fixation in Bouin's fluid the mammae were dissected off from the skin, muscle and covering fascia with the aid of a dissecting microscope magnifying 7 and 14 times. Nerve fibres were removed and some of the large blood vessels. These stripped mammae were then immersed overnight in 50 per cent alcohol, and next stained for about 4 hours in 1 part of Ehrlich's acid haematoxylin freshly diluted with 3 parts of a 2 per cent aqueous solution of acetic acid. After differentiating for about 10 minutes in acid alcohol (3 per cent HCl in 50 per cent alcohol) the stained glands were rendered blue or blue-black in several changes of 50, then 70 per cent alcohol for as many hours or days as were required to develop a deep colour. They were next dehydrated and cleared in the usual manner. While in the first change of clearing agent (xylol) notes were made by the author after examining all 10 mammae or fat pads with the binocular dissecting microscope. Some were selected for making permanent mounted preparations and for storage in paraffin. The remainder were discarded.

### RESULTS.

*R3X females deprived of their ovaries and of the milk-borne tumour agent.*

Twenty days after the application to the skin of 400 microgrammes of keto-hydroxyoestrin, 0·1 per cent dissolved in acetone, the duct system had grown out and branched extensively (Fig. 3). The ducts were distended and filled with milky secretion, which rendered the main branches and many smaller ones visible to the naked eye in all 10 mammary glands. There was also a pseudo-lobular alveolar differentiation with great multiplication of acini arranged in clusters in all 10 mammary glands. The large clusters could also be seen macroscopically as milky white lobed structures. Many of the acini composing them were ballooned out with secretion (Fig. 3). A similar response was given by the maximum dose of 400 Y of $\alpha$-oestradiol repeated at 20 days' interval (total 800 Y) in 15 out of 15 mice which were examined *in vivo*. The most noticeable difference after the larger dose was outgrowth in every mouse into interscapular fat pads and possibly more secretion (Fig 4).

These responses are atypical and pathological. They differ from changes in the normal female mouse in oestrus (Cole, 1933; Turner and Gomez, 1933) and from responses in normal mice artificially treated with oestrogens (Turner and Gomez, 1934) in that lobular-alveolar differentiation and secretion occur in addition to duct outgrowth. Instead of a single response by duct outgrowth, there is a triple response to the one hormone. It is like a pseudo-pregnancy response. It is pathological in that it is irregular, distorted by distension, and patchy in distribution within the glands. In extent it did not exceed the limits of outgrowth attained during normal pregnancy. It is pathological also in that the degree of differentiation is suggestive of a 9th–12th day pregnancy while the milky secretion is voluminous. Male glands reacted to larger doses in a similar manner. They were often more irregular in arrangement of ducts and acini. In normal stock mice and in rats also the response of the mammae to spontaneously secreted or to injected oestrogens is by simple duct outgrowth (Fig. 7). Lobular-alveolar growth requires the stimulus of pregnancy or of artificially applied progesterone in addition to oestrogen (Mixner and Turner, 1943). Normal unmated female mice have no luteal phase in the ovarian cycle and are said to secrete no luteal hormone. The atypical oestrogen response of R3X and Simpson mice is a hormone conditioned hypertrophy and hyperplasia, including adenosis and secretion by the glandular cells. That it is atypical, even among inbred strains, is shown by comparison with C57 (black) and Strong AX virgin female mammae after identical treatment. No dose of oestrogen was found that gave a similar response in C57 (black) females or males or in Strong A or AX females or males. Continued treatment over a long period of time sometimes resulted in slight local acinus formation in Strong A mice. At the age of one month no response was observed in R3X females save for a tendency of the end bulbs to canalize.

The minimum dose of oestrogen required to reveal this capacity for atypical responses in R3X mice and the time after treatment when it first appears have not yet been found. This atypical response is the cystic disease of mouse mammae first described by Haaland (1911) as occurring spontaneously in mixed stock (though in these animals a milk-borne agent may also have been present), and later by Gibson (1930) in inbred strains. It was first reproduced experimentally in mixed stock mice in 9 out of 11 spayed females by Goormaghtigh and Amerlink (1930), who compared it with Reclus disease in the human breast. Experiments here recorded provide the additional information that the response can be provoked independently of the presence of milk-borne tumour agent, and confirm opinions previously expressed that it is a strain characteristic. It is of interest that in a small series of virgin and breeding R3X females cystic distension has never been observed to occur spontaneously (in 25 out of 25 examined).

The atypical response might be due either to local differences in sensitivity of the end bulbs of various strains of mice or to the response in the pituitary gland, where, according to Gomez and Turner (1937 ; Gomez, Turner and Reece, 1937) the effective mammogenic hormones are produced, or to a combined response in both organs. Some light on this problem was gained from experiments similar to those here described using the double dose of 400 microgrammes of oestradiol on spayed F.1 hybrid females of the C57 x R3X cross. The curious observation was made that a similar lobular-alveolar response was provoked in all the mammae, but it was localized in approximately half of each gland. The other half responded typically like the C57 (black) parent's gland (Fig. 8). These results suggest that the atypical response is due in part at least to variation in local sensitivity of ducts and end bulbs, and not solely to a strain difference in the pituitary response.

In the absence of ovaries and of any further application of hormone the characteristic but atypical reaction persisted in R3X mice for about ten weeks after the beginning of the experiment, using 800 microgrammes of oestradiol.

Regression of the whole of the recent acinar and pseudo-lobular-alveolar development started after about the 10th week, and was complete in most of the mice between the 16th and 20th week. The mice were then 6 to 7 months old. The exact time taken for complete regression varied with the degree of the original response, which in its turn varied slightly in individual mice. Complete regression was indicated by total disappearance of all lobules, acini and secretion. Bare, shrunken, almost linear ducts remained (Fig. 5). Details of the number of mice examined in this way and of complete or incomplete regression of the hormone response are given in Table I. Some of the animals killed at 16 weeks revealed remnants of atrophic pigmented acini usually in small groups at the periphery of the 4th mammae, probably at the site of former large clusters. Fig. 9 is representative of these imperfections of involution. Sometimes detached ghost acini remained unabsorbed (Fig. 10). No inflammatory or metaplastic nodules were encountered.

In the course of these experiments the possibility had to be considered that in the females some other sources of oestrogens might be stimulated by ovariectomy and after cessation of the action of artificially applied hormone to produce oestrogens, as Smith and Bittner (1945) found in the C3H strain. That no such stimulation had occurred was proved by making vaginal smears during the last two to three weeks of the experimental period in 11 out of 20 mice, and by inspection of uterus and oviducts at autopsy. In no case was there a return of oestrus or failure of uterus and oviducts to atrophy.

### *R3 Virgin Females Deprived of their Ovaries but Containing the Milk-borne Tumour Agent.*

Early changes in the mammae in response to ovariectomy and oestrogen treatment (ketohydroxyoestrin and oestradiol) were similar to those described in the same strain deprived of the tumour agent (32 out of 32 mice). Differences were detectable in the intervening period, but it will be convenient first to contrast the final results at 16 to 20 weeks in the two sets of mice. Proliferation due to hormone action regressed at a similar rate and was complete, so far as this can be judged in the presence of the tumour agent, at some time between the 16th and 20th weeks of experiment. Regression was expressed in the greater part of all mammae by disappearance of lobules and alveoli and by the presence of a shrunken duct system. In the majority of mammae, however, there persisted localized nodular areas of proliferation of acini on the duct system (Fig. 6). These were found in all of 28 mice and, in addition, 11 out of 40 mice killed before the 8th month of life bore mammary adenocarcinomas. The earliest of these appeared at four-and-a-half months old. The nodules of proliferation occurred on otherwise atrophic duct systems, giving a picture similar to that described by Gardner *et al.* (1939). By the vaginal smear technique, oestrus cells were seen on one occasion in each of 2 mice out of 32 examined. In 30 mice oestrus cycles or cells did not recur.

The contrast between the mammae in the two sets of mice at 16 to 20 weeks was striking. There was complete absence of adenomas in those mice which had been deprived of the milk-borne tumour agent, whereas in mice of the original R3 strains carrying milk agent adenomas were numerous in nearly all the glands of every mouse. Fig. 5 and 6 are representative of 200 mammae from the R3X females and 280 from those carrying the agent. A few imperfections due to incomplete regression did not cause confusion, and will be referred to in this connection again.

The conclusions were drawn that the foci of hyperplasia (adenomas) in the R3 females carrying tumour agent, were due in respect of their origin and persistence to the presence of a milk-borne tumour agent, presumably identical with or a variant of Bittner's agent, and that this agent had been forced into activity by the large dose of hormone. These nodules are considered to be experimentally produced counterparts of those that occur spontaneously in all R3 females after the age of 8 to 9 months. They are similar to those described in great detail, and finely illustrated by Gardner (1942) in other strains. Gardner demonstrated the persistence of spontaneous pregnancy-activated adenomas in the absence of further hormone stimulation in hypophysectomized breeder females. The present experiments provide a simple method of forcibly stimulating adenomas and of revealing their dependence on the milk-borne tumour agent.

A few more descriptive details that are characteristic of R3 adenomas may be added to Gardner's comprehensive accounts. Both spontaneous and forcibly activated R3 adenomas are of two main varieties, compact (Fig. 6) and diffuse (Fig. 11) clusters of acini connected by shorter or longer lengths of duct. Some of the former appear to lack duct formation altogether. Some of the latter may be largely composed of ducts, similar to one of Gardner's, or even of end-bulbs. Many of the diffuse loosely constructed adenomas contain secondary foci of more active proliferation (Fig. 11 and 12), indicating perhaps sites of greater vigour of the stimulating agent, and providing evidence for the existence previously suggested of variants of the agent. These secondary foci were found frequently in both spontaneous and artificially activated adenomas, including many in males treated with hormone. They too were independent of ovarian hormone for maintenance once they had been activated. No inflammatory or metaplastic nodules were encountered.

The earliest date at which adenomatous proliferation was found was at 21 days after a single dose of 200 microgrammes of ketohydroxyoestrin (Fig. 14). This finding was not constant at 21 days. Adenomas were found in 4 out of 4 mice at 9 weeks after 800 microgrammes given in 2 doses of 400 microgrammes each at 20-day intervals, applied to spayed females at approximately 56 days old. At these dates the hormonal response in the mammae was still in existence, and the interpretation of an adenomatous response was open to question (Fig. 13). The differentiation between focal hyperplasia or adenosis caused and maintained by hormone alone and adenomatous nodules caused and maintained by milk-borne tumour agent plus hormone is possible in some nodules only. In others no certain decision can be reached. The chief difference in the two kinds of clusters of acini rests on their spatial arrangement and relative regularity. The hormone induced hyperplasias tend to lie in one plane ; the combined response of hormone and milk-borne tumour agent is exceedingly irregular in comparison and lies in all dimensions (Fig. 3 and 13). When, as in one adenomatous nodule seen at 3 weeks (Fig. 14) numerous end bulbs were observed arising from a cluster of acini, there can be no doubt of the pathological and atypical origin of the proliferation due to stimulation other than by hormones. End bulbs, though sometimes multiple, are normally situated at the tips only of growing ducts

(Cole, 1933). This is also the case when artificially supplied hormones are acting alone.

The smallest total dose of oestrogen which constantly activated the milk-borne tumour agent into pathological activity as shown by adenoma production was 50 microgrammes of ketohydroxyoestrin given through the skin in a small series of mice (Table I).

*R3X Males Deprived of the Milk-borne Tumour Agent.*

The rudiments of mammae of males derived from the same sublines as the females and treated with larger doses of ovarian hormones responded in a similar manner. An average of 6 mammary glands per mouse developed from rudiments and all examined revealed the atypical response characteristic of females of this strain. Irregular lobules were seen after cutaneous application of 800–1200 microgrammes of oestradiol with or without 1500 microgrammes of progesterone. Regression required 6 to 7 months to become complete, leaving bare atrophic ducts (Table II).

*R3 Males Containing Milk-borne Tumour Agent.*

When additional time had been allowed for regression of hormone response, results were similar, and almost as regular as in spayed females containing milk-borne tumour agent. Either oestrogen alone (1200 microgrammes) or combined with one dose of progesterone (1,500 microgrammes) was sufficient to stimulate the growth of adenomas (Table II). Adenomas were found in 38 out of 40 male mice, but were not so numerous in individuals as in the females. Nine of the males developed malignant tumours by this treatment in 5 months.

Twelve males which were castrated before treatment with hormone developed adenomas but no malignant tumours. The hormone dosage used was better tolerated by all the male mice than by the more usual protracted method. Results were not better in respect of stimulation when progesterone was added than in experiments in which oestradiol alone was used (Table II) but a larger number of malignant growths was seen.

*Other Varieties of Mice.*

The same methods were applied in preliminary tests to C3H, Strong A, Simpson and C57 (black) strains. Results are summarized in Table III. The fact that mammae of Strong A females respond normally to oestrogen, that is by duct outgrowth only, indicates that the atypical pseudo-lobular-alveolar response probably does not represent a structural basis of the hereditary factor underlying susceptibility to pathological activity of milk-borne tumour agent.

Since it might be suggested that the lobular-alveolar response is an expression of a mutation in some highly inbred strains, a few stock mice obtained from a dealer were examined by the same technique as for hormone response. These were got from a town where, so far as one knew, no inbreeding of pure lines was being carried on. This precaution was taken to avoid mice that originated from pure line laboratory bred strains. One out of 11 of these mongrel mice responded with early widespread pseudo-lobular-alveolar differentiation and secretion. In degree the distension and proliferation were less than in some of the inbred strains, but in distribution and kind the reactions were very similar. This indicates that if the reaction is based on a mutation, then the mutation can occur also under relatively natural conditions.

The test for activation of milk-borne tumour agent has been applied to reciprocal hybrids and the results are incomplete. They indicate so far that hybrids are suitable experimental material for the present purpose (Table III).

COMMENT.

The work here recorded confirms the opinion already widely held that hyperplastic nodules in the mammae of mice provide evidence of pathological activity of the milk-borne tumour agent, and shows how this activity can be forced and detected sooner than by the spontaneous appearance of mammary tumours. The presumption is that the agent provoking nodular hyperplasia and the tumour agent are the same or of like nature, because in both cases the response is by cell proliferation. Further work is required to settle the degree of similarity. The two objections to accepting nodular hyperplasia as evidence of activity of the agent have been met. The criteria proposed as bases for the validity of the test were twofold. They required the absence of nodular hyperplasia (adenomas), and of all other signs of acinus formation in the absence of the agent and absence of hormone. They required the development of adenomas in involuted mammae in the presence of the agent and absence of hormone. As has been recorded, perfection in respect of complete absence of acini required in the first criterion was attained in 15 out of 20 females; in 5 others killed at an earlier date acini were found which were regarded as being, at the time of death of the mice, in process of regression. There was, in the authors' opinion, no reasonable possibility of confusing these surviving acini with adenomas. The surviving acini were grouped along the periphery of the gland (Fig. 9) in a manner bearing no resemblance to adenomas (Fig. 6). Their shrunken atrophic character was apparent. It is probable that the provocative doses of hormone were larger than needed for the purpose, and that given the same time for involution to take place a smaller dose would have been succeeded by complete disappearance of acini. Alternatively a longer interval must be allowed for the larger dose. Regression of hormone response was more often complete in males, probably owing to the longer time interval allowed for it (Table II). Success in respect of the second criterion was attained in 28 out of 28 females and in 28 out of 30 males.

SUMMARY.

1. Variations in response to oestrogenic hormones in the mammae of spayed females of several strains of mice have been confirmed. They have also been found in mice from a dealer. The variations consist of pseudo-lobular-alveolar differentiation and secretion of milky fluid in addition to the normal response of duct outgrowth. The response is a pathological hyperplasia or adenosis of the mammary gland. It is independent of the presence of the milk-borne tumour agent. In the absence of further oestrogenic stimulation in spayed mice this pseudo-lobular-alveolar response regressed, leaving bare shrunken ducts.

2. A second type of response was elicited in R3 and C3H mice carrying the milk-borne tumour agent. This second response was identified with certainty as caused by the milk-borne tumour agent (Bittner's or a variant of it) only when all other formative agents were excluded. This was done by spaying and providing a limited dose of hormone to provoke pathogenic activity of the agent. It was characterized by the appearance of adenomas in otherwise involuted mammae. It is suggested that these adenomas are to be regarded as evidence of activity of milk-borne tumour agent when all other formative agents have been excluded, whether they occur spontaneously or are forcibly stimulated.

### REFERENCES.

ANDERVONT, H.—(1945) *In* a Symposium on Mammary Tumours in Mice. Washington, D.C., p. 134.

APOLANT, H.—(1906) *Arb. a. d. Königlichen Inst. exp. Therap., Frankfürt A.M.*, **1**, 7.

BITTNER, J. J.—(1936) *Science*, **84**, 162.—(1939) *Publ. Hlth. Rpts.*, **5**, 1590.—(1943) *Cancer Res.*, **3**, 441.—(1945) *In* the A.A.A.S. Research Conference on Cancer. Washington, D.C., p. 63.

*Idem*, HUESBY, R. A., VISSCHER, M. B., BALL, Z. B., AND SMITH, F.—(1944) *Science*, **99**, 83.

BONSER, G. M.—(1936) *J. Path. Bact.* **42**, 169.

BURROWS, H.—(1936) *Ibid.*, **42**, 161.—(1945) 'Biological Actions of Sex Hormones,' Cambridge, p. 330.

COEN, E.—(1887–88) *Beitr. Z. path. Anat. u. Physiol.*, **2**, 85.

COLE, H. A.—(1933) *Proc. Roy. Soc.*, B., **114**, 136.

GARDNER, W. U.—(1942) *Cancer Res.*, **2**, 476.

*Idem*, DIDDLE, A. W., ALLEN, E., AND STRONG, L. C.—(1934) *Anat. Rec.*, **60**, 457.

*Idem*, SMITH, G. M., AND STRONG, L. C.—(1935) *Proc. Soc. exp. Biol. Med.*, **33**, 148.

*Idem*, STRONG, L. C., AND SMITH, G. M.—(1939) *Amer. J. Cancer*, **37**, 510.

GIBSON, L. M.—(1930) *J. Cancer Res.*, **14**, 570.

GOMEZ, E. T., AND TURNER, C. W.—(1937) *Bull. Mo. agric. Exp. Sta.*, No. 259.

*Iidem* AND REECE, R. P.—(1937) *Proc. Soc. exp. Biol. Med.*, **36**, 286.

GOORMAGHTIGH, N., AND AMERLINK, A.—(1930) *Etud. Cancer*, **19**, 527.

v. GULIK, P. J., AND KORTEWEG, R.—(1940) *Nederl. Akad. v. Wetensch.*, **43**, 891.

HAALAND, M.—(1911) 'Fourth Scientific Rep. Imp. Cancer Res. Fund, London,' p. 1.

HUESBY, R. A., AND BITTNER, J. J.—(1946) *Cancer Res.*, **6**, 240.

KIRSCHBAUM, W., WILLIAMS, W. L., AND BITTNER, J. J.—(1946) *Ibid.*, **6**, 354.

LACASSAGNE, A.—(1934) *C.R. Soc. Biol., Paris*, **115**, 937.

LATHROP, A. E. C., AND LOEB, L.—(1916) *J. Cancer Res.*, **1**, 1.

MACKENZIE, I., AND ROUS, P.—(1941) *J. exp. Med.*, **73**, 391.

MIXNER, J. P., AND TURNER, C. W.—(1943) *Bull. Mo. Agric. Exp. Sta.*, No. 378, p. 20.

ROUS, P., AND KIDD, J. G.—(1941) *J. exp. Med.*, **73**, 365.

SHIMKIN, M. B.—(1945) *In* a Symposium on Mammary Cancer in Mice. Washington, D.C., p. 97.

SMITH, F. W., AND BITTNER, J. J.—(1945) *Cancer Res.*, **5**, 588.

TURNER, C. W., AND GOMEZ, E. T.—(1933) *Bull. Mo. agric. Exp. Sta.*, No. 182.—(1934) *Ibid.*, No. 206.

WOGLOM, W. H.—(1945) *Cancer Res.*, **5**, 576.